# Metabonomics Study on Naotaifang Extract Alleviating Neuronal Apoptosis after Cerebral Ischemia-Reperfusion Injury

**DOI:** 10.1155/2022/2112433

**Published:** 2022-03-14

**Authors:** Dehong Xu, Qidi Ai, Xiaoqing Chen, Zhaoguo Wang, Hongda Wei, Luobing Zhou, Zhigang Mei, Jinwen Ge

**Affiliations:** ^1^College of Integrated Traditional Chinese and Western Medicine, Hunan University of Chinese Medicine, Changsha, Hunan 410208, China; ^2^Laboratory of Biological Engineering, College of Pharmacy, Hunan University of Chinese Medicine, Changsha, Hunan 410208, China; ^3^School of Medicine, Shaoyang University, Shaoyang, Hunan 422000, China

## Abstract

Naotaifang extract (NTE) is a clinically effective traditional Chinese medicine compound for cerebral ischemia-reperfusion injury. Although NTE can achieve neuroprotective function through different mechanisms, the pharmacodynamic substances of NTE corresponding to these mechanisms have rarely been reported. Alleviating or inhibiting neuronal apoptosis is an important way to achieve neuroprotection. Accordingly, this study has evaluated the effects of NTE on alleviating neuronal apoptosis after cerebral ischemia-reperfusion injury from two levels of cells and tissues. Meanwhile, the serum pharmacochemistry of NTE was analyzed by high performance liquid chromatography-tandem mass spectrometry (HPLC-MS/MS) with the guidance of Chinmedomics. The results included three aspects: (1) NTE could significantly alleviate neuronal apoptosis caused by in vitro cellular models and in vivo animal models; (2) a total of 21 serum differential metabolites was discovered, including adenosine, inosine, ferulic acid, calycosin, salidroside, 6-gingerol, 2-methoxycinnamaldehyde, and so on; (3) the metabolic pathway regulated by NTE was mainly purine metabolism. From these results, it can be concluded that alleviating neuronal apoptosis by NTE after cerebral ischemia-reperfusion injury is one of the important mechanisms to achieve neuroprotection. The pharmacodynamic substances of NTE for alleviating neuronal apoptosis on the one hand are related to components directly absorbed into blood, such as ferulic acid, calycosin, salidroside, 6-gingerol, and 2-methoxycinnamaldehyde and on the other hand are also closely linked to its indirect regulation of purine metabolism in the body to produce adenosine and inosine. Therefore, our research not only identified the main pharmacodynamic substances of NTE that alleviated neuronal apoptosis but also provided a methodological reference for studying other neuroprotective effects of NTE.

## 1. Introduction

Stroke is a disease with cerebral ischemia or hemorrhage as its main clinical symptom, which can be divided into ischemic strokes (i.e., cerebral infarction) and hemorrhagic strokes (i.e., cerebral hemorrhage). Cerebral infarction dominates the clinical stroke events [1, 2] and seriously endangers people's life and health due to its acute onset, high incidence, high disability rate, and high mortality rate [2, 3]. When cerebral ischemic stroke occurs, timely recovering reperfusion is the main way to treat the disease, but the recovery of blood-oxygen supply often aggravates oxidative stress and inflammatory reaction, which lead to various forms of cell death and ischemic tissue injury [4]. Of different cell death modes, apoptosis is the key mechanism of neuronal death in the ischemic area [5], so inhibiting neuronal apoptosis is an important way to achieve neuroprotection and alleviate cerebral ischemia/reperfusion injury (CIRI).

Naotaifang extract (NTE) is a clinically effective traditional Chinese medicine (TCM) compound for ischemic stroke, and its formula consists of four drugs including Astragalus root (Huangqi), Rhizoma Chuanxiong (Chuanxiong), Lumbricus (Dilong), and Bombyx Batryticatus (Jiangcan) [6]. Compared with the positive drug, NTE was better than nimodipine in terms of healing rate, percent reduction in clinical neurological function, and improvement in blood flow abnormalities [7]. Further studies on the NTE have showed that it played functions in ischemic stroke through various molecular mechanisms. Huang et al. reported that NTE could affect the expression of nuclear factor-E2-related factor 2 (Nrf2), heme oxygenase-1 (HO-1), and hephaestin in the hippocampus of CIRI rats [8]. He et al. discovered that the protective effect of NTE on ischemic brain tissue was to reduce the excessive accumulation of glutamate in synapses of neurons by improving the function of glutamate transporters, which eventually reduced excitotoxicity of glutamate [9]. More researches indicated that NTE had protective effects on neuron ferroptosis induced by acute cerebral ischemia in rats [6, 10]. The discoveries of the previously mentioned mechanisms, on the one hand, showed that NTE had two characteristics of complex targets and diverse pathways in treating ischemic stroke, and on the other hand, they also put forward a question for pharmaceutical researchers to answer; that is, what were the pharmacodynamic substances that caused NTE to produce corresponding mechanisms of action?

Pharmacodynamic substances of TCM refer to chemical constituents contained in TCM that can exert the clinical efficacy of drugs [11]. The complexity and multicomponent synergy of TCM have made the pharmacodynamic substances of many herbal medicines, especially compound medicines, remain in a “black box” state. In order to open the “black box,” different researchers had successively applied various methods to study the pharmacodynamic substances of TCM, of which the most systematic methodology is Chinmedomics [11]. This method organically combines serum pharmacochemistry of TCM with metabonomics to search for the pharmacodynamic substances of TCM by analyzing the transport constituents in the serum after oral administration of TCM [12], which is a practical, simple, and quick research method that conforms to the theoretical system of TCM. In the practical application of Chinmedomics to study the pharmacodynamic substances of TCM, the high-throughput and high-sensitivity liquid-mass spectrometry (LC-MS) detection technique is often used as the preferred method to analyze transport constituents in serum samples accurately [13].

To summarize, guided by Chinmedomics, this study used LC-MS to analyze the serum pharmacochemistry of NTE in the hopes of identifying the pharmacodynamic substances of NTE alleviating neuronal apoptosis after CIRI, as well as providing a methodological reference for studying the pharmacodynamic substances of NTE for other neuroprotective effects.

## 2. Materials and Methods

### 2.1. Drugs

Ninety g Astragalus root (Huangqi), 22.5 g Rhizoma Chuanxiong (Chuanxiong), 33.75 g Lumbricus (Dilong), and 33.75 g Bombyx Batryticatus (Jiang Can) were weighed, then soaked in 5 times of water for 15 min, and decocted for 30 min. The residue was steeped in three times of water for 15 min after the liquid medicine was filtered using gauze and then decocted for 30 minutes. For later usage, the two decoctions are combined, filtered, and concentrated to 2 g crude drug/mL liquid medicine. The normal saline was used to dilute NTE to required concentration.

### 2.2. Sprague Dawley (SD) Rats

Healthy adult male SD rats, 80 ± 5 days old and weighing 220–250 g, as study subjects were provided by the Hunan Slack Jingda Experimental Animal Co., Ltd (Hunan, China). Rats were maintained in SPF laboratory of Experimental Animal Center of Hunan University of Traditional Chinese Medicine, given standard feed and clean water, and kept at 25°C room temperature and about 50% relative humidity. Feeding was stopped 12 hours before the experiment, but water was still given. All animal experiments were approved by the Medical Animal Ethics Committee of Hunan University of Traditional Chinese Medicine (Changsha, China, ethic number: LLBH-202101190004) on 19/1/2021.

### 2.3. Effects of NTE on PC12 Cells after Recovery from Oxygen-Glucose Deprivation

#### 2.3.1. Grouping of SD Rats

25 SD rats were randomly divided into five groups: NTE low-dose group (10 g/kg, NTE-L), NTE medium-dose group (20 g/kg, NTE-M), NTE high-dose group (30 g/kg, NTE-H), Edaravone group (10 mg/kg, E), and normal saline group (NS).

#### 2.3.2. Preparation of Drug-Containing Serum and Drug-Free Serum

NTE was administered intragastrically, while edaravone (batch number: 190512, Fujian TianQuan Pharmaceutical Co., Ltd., China) was administered intraperitoneally. The preparation process of drug-containing serum, including NTE-L serum, NTE-M serum, NTE-H serum, and E serum, is briefly described as follows. Different-dose NTE and edaravone were administrated to rats once a day for seven consecutive days, respectively. Blood was drawn from the jugular vein and deposited in sterile centrifuge tubes, which were then centrifuged at 4000 r/min in a chilled centrifuge. The supernatant was extracted, inactivated for 30 minutes in a 56°C thermostat water bath, sterilized with a 0.22 m microporous membrane, and then kept at −80°C for subsequent use. The production of drug-free serum was identical to that of drug-containing serum, with the exception that, in SD rats, the same volume of normal saline was used instead of NTE.

#### 2.3.3. Culture, Grouping, and Processing of PC12 Cells

According to literature [14], the oxygen-glucose deprivation (OGD) state during cerebral ischemia was simulated by glucose-free medium containing cobalt dichloride (CoCl_2_), while the reperfusion state with oxygen-glucose supply recovery was simulated by glucose medium without CoCl_2_. PC12 cells (frozen in our laboratory), a commonly used nerve cell strain, were cultured in Dulbecco's Modified Eagle Media (DMEM) medium (Sangon Biotech, China) containing 10% fetal bovine serum (SERANA, Brandenburg, Germany) and 1% double antibody at 37°C, 5% CO_2_, and saturation humidity, using a carbon dioxide incubator (Thermo fisher scientific, Massachusetts, USA). When the cells were cultured to exponential phase, they were dissociated with trypsin digestion (HycloneTM, Utah, USA), collected, and then seeded in a 24 well plate at a concentration of 10^5^ cells/well. PC12 cells were divided into six groups, each with three wells: control group (C), recovery from OGD group (R), edaravone group (E), NTE low-dose group (L), NTE middle-dose group (M), and NTE high-dose group (H). After incubation for overnight, the cells were treated as shown in Table 1.

#### 2.3.4. Detection of Relative Cell Viability

Ten *μ*L of cell counting kit-8 (CCK-8) reagent (Sangon Biotech, China) was added to each well, and the plates were incubated in the dark for 1 h at 37°C. Subsequently, the absorbance of solution was measured at 450 nm by a Varioskan Flash plate reader (Thermo fisher scientific, Massachusetts, USA). The relative cell viability was calculated using the following equation: (A_test_-A_blank_)/(A_control_-A_blank_), where “A_blank_” is the absorbance of wells containing only medium and CCK-8 solution, “A_control_” is the absorbance of the group C, and “A_test_” is the absorbance of the groups R, E, L, M, and H.

#### 2.3.5. Detection of Apoptosis

The cultivated cells were digested by trypsin, washed twice with phosphate buffer saline (PBS), and stained according to the Annexin V-FITC/propidium iodide (PI) staining kit instruction manual (YEASEN Biotech, China). The staining state of cells and the percentage of Annexin V and PI double positive cells were analyzed by flow cytometry.

### 2.4. Metabolomic Analysis of NTE Chemical Components Absorbed into Blood

#### 2.4.1. Grouping of SD Rats

The SD rats were divided into two groups with 16 animals in each group: NTE high-dose group (30 g/kg, NTE-H) and normal saline group (NS), respectively.

#### 2.4.2. The Model of Focal Cerebral Ischemia-Reperfusion Injury in Rats

A rat model of middle cerebral artery occlusion (MCAO) was established by referring to the Longa method and improving it [15]. MCAO was induced by using the intraluminal filament technique. Right common and external carotid arteries were ligated and the internal carotid artery was closed. A fish wire (*d* = 0.28 mm) was advanced through the right internal carotid artery to the origin of the MCA. Ischemia/reperfusion (I/R) phenomenon was caused by withdrawing the fish wire after two hours of embolization. In the process of establishing the CIRI model, rats were anesthetized with 1.4% isoflurane.

#### 2.4.3. Brain Tissue Preparation

The rats were given the same volume of NTE and normal saline for the first time 24 h after awakening from anesthesia. NTE-H and NS were administrated to rats once a day for seven consecutive days, respectively. One hour after the last administration, 10 rats in each group were anesthetized with isoflurane, of which five rats were used for 2,3,5-triphenyltetrazolium chloride (TTC) staining, and the other five rats were used for terminal-deoxynucleotidyl transferase/(TDT-) mediated nick end labeling (TUNEL) detection. For TTC-stained rats, the brains were taken out by decapitation, frozen in a refrigerator at −20°C for 30 min, and then taken out from the refrigerator when it was slightly hardened. Finally, the two hemispheres of the brain were cut into coronal sections with a thickness of 1.5 mm using a microtome. For TUNEL-detected rats, the brain tissue was perfused with 0.9% normal saline and then with 4% paraformaldehyde. Finally, the brain tissue was embedded in paraffin and sliced.

#### 2.4.4. TTC Staining

The brain slices were incubated in 2% TTC (Sangon Biotech, China) at 37°C for 30 minutes, turned over every 5 minutes, and then washed three times with ddH_2_O. Each brain slice was analyzed by Image-Pro Plus software to measure its infarct area and total area. The infarct volume of each layer is the product of the infarct area and the thickness of the layer. The total infarct volume is the sum of the infarct volumes at each layer. The infarct volume percentage was calculated using the following equation: total infarct volume/total brain volume × 100%.

#### 2.4.5. TUNEL Detection

First, brain slices were incubated with methanol containing 0.2% H_2_O_2_ for 0.5 h to block endogenous peroxidase activity. Then, brain slices were treated with TUNEL reaction mixture (Sangon Biotech, China) and kept in an incubator at 37°C for 60 minutes. Finally, after routine dehydration, transparence, and sealing, cells were randomly selected from three nonoverlapping visual fields of cerebral cortex under high magnification (400x) to count the average number of TUNEL positive cells (brown-yellow granules in the nucleus).

#### 2.4.6. Preprocessing of Serum Samples

Blood samples were collected from the remaining six modeled rats in each group through the jugular vein to be placed in sterile centrifuge tubes and centrifuged at 4000 r/min by refrigerated centrifuge. The supernatant was taken, of which the serum from NTE-H and NS were labeled with NTE-P and NS-P, respectively. 100 *μ*L serum from each group was taken into a 1.5 mL centrifuge tube with 300 *μ*L methanol (containing 1 ppm 2-chlorophenylalanine). After swirling for 2 min, all of centrifuge tubes were incubated in a refrigerator at −20°C for 0.5 h and then centrifuged at 12000 r/min and 4°C for 10 min. The 200 *μ*L supernatant from each group was transferred to a new 1.5 mL centrifuge tube and incubated it at −20°C for 0.5 h. After centrifuging at 12000 r/min and 4°C for 15 min, the supernatant was taken into the sample bottle for LC-MS/MS analysis.

#### 2.4.7. Liquid Chromatography Conditions

Metabolomics analyses were performed on an Agilent 1290 Infinity LC ultrahigh pressure liquid chromatography (UHPLC) (Agilent, Palo Alto, USA) equipped with an electrospray ionization source operating in positive and negative ion modes.

For the metabolomics analysis, a Waters T3 C_18_ column (2.1 × 100 mm, 1.8 *μ*m, Waters, USA) was used. The column was maintained at 40°C and eluted at a flow rate of 0.35 mL/min. The mobile phase was composed of A (0.04% acetic acid in water) and B (0.04% acetic acid in acetonitrile). The process of linear gradient elution was as follows: 0∼10 min, 5%∼95% B; 10∼11 min, 95% B; 11∼11.1 min, 95%∼5% B; 11.1∼14 min, 5% B. The auto sampler was maintained at 4°C and the injection volume was 2 *μ*L.

#### 2.4.8. Q-TOF Mass Spectrometric Conditions

The mass spectrometer (MS) was Q-TOF/MS-6545 system (Agilent, Palo Alto, USA) equipped with an electrospray (ESI) as ionization source in positive (ESI+) and negative (ESI−) ion modes. The detection parameters of Q-TOF mass spectrometry were set as previously described [16], which were made the appropriate modifications. The MS properties were set as follows: scan range, *m*/*z* of 50–1000 Da; product ion scan *m*/*z* range, 25–1000 Da; time of flight (TOF) MS scan accumulation time, 0.2 s/spectra; product ion scan accumulation time, 0.05 s/spectra; ion source gas1 (Gas1), 50 psi; ion source gas2 (Gas2), 80 psi; curtain gas (CUR), 25 psi; source temperature, 500°C; ion spray voltage floating (ISVF), ±5000 V; declustering potential (DP), ±80 V; collision energy, 35 ± 15 eV. MS/MS data were acquired in the information dependent acquisition (IDA) mode and using high-sensitivity modes. The settings of IDA were as follows: exclude isotopes within 4 Da, candidate ions to monitor per cycle, 10.

#### 2.4.9. Data Processing and Statistical Analysis

The original data file obtained by LC-MS analysis was firstly converted into mzML format by ProteoWizard software. Peak extraction, alignment, and retention time correction were performed by XCMS program. After installing corresponding software packages (such as pheatmap 1.0.12, MetaboAnalystR 1.0.1), the preprocessed data were statistically analyzed by R programming language, including orthogonal partial least square-discriminate analysis (OPLS-DA), volcano map analysis, and metabolic pathway analysis.

#### 2.4.10. Screening and Identification of Differential Metabolites

In this study, variable importance in projection (VIP)≥1, Student's *t*-test *p* < 0.05, and fold change (FC) ≥2 or ≤0.5 were used as screening criteria for differential metabolic data. The METLIN database was used to identify potential differential metabolites candidates based on their MS signature and tandem mass spectrometry (MS/MS) spectra, as well as eventual contaminants. Identification of potential differential metabolites was carried out by searching METLIN (http://metlin.scripps.edu/), HMDB (http://www.hmdb.ca/), KEGG (http://www.genome.jp/kegg/), MassBank (http://www.massbank.jp/), LIPIDMAPS (http://www.lipidmaps.org/), and ChemSpider (http://www.chemspider.com) using exact molecular weights or MS/MS fragmentation pattern data and literature search to identify the affected metabolic pathways and facilitate further biological interpretation.

### 2.5. Statistical Analysis

Data analysis was performed using SPSS 18. All data are presented as means ± standard deviation, and *n* is the number of independent experiment cells. Significant difference was compared by Student's *t*-test.

## 3. Results

### 3.1. The Effect of NTE on PC12 Cells Viability after Recovery from OGD

**Figure 1:**
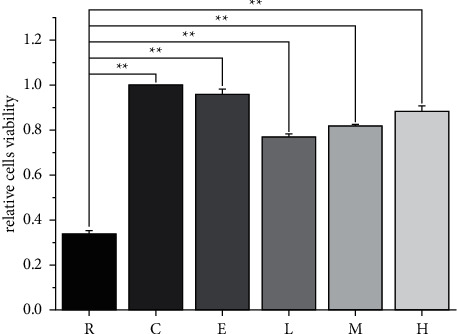
Detection of PC12 cells viability after recovery from OGD. ^*∗∗*^ means *p* < 0.01. Data are presented in the form of means ± standard deviation (*n* = 3). Control group (C); recovery from OGD group (R); edaravone group (E); NTE low-dose group (L); NTE middle-dose group (M); NTE high-dose group (H).

After recovery from OGD, the PC12 cells viability without drug treatment decreased significantly, while the positive drug edaravone and different concentrations of NTE could significantly increase the viability of PC12 cells. The effect of NTE on PC12 cells was concentration-dependent (Figure 1).

### 3.2. The Effect of NTE on PC12 Cells Apoptosis after Recovery from OGD

**Figure 2:**
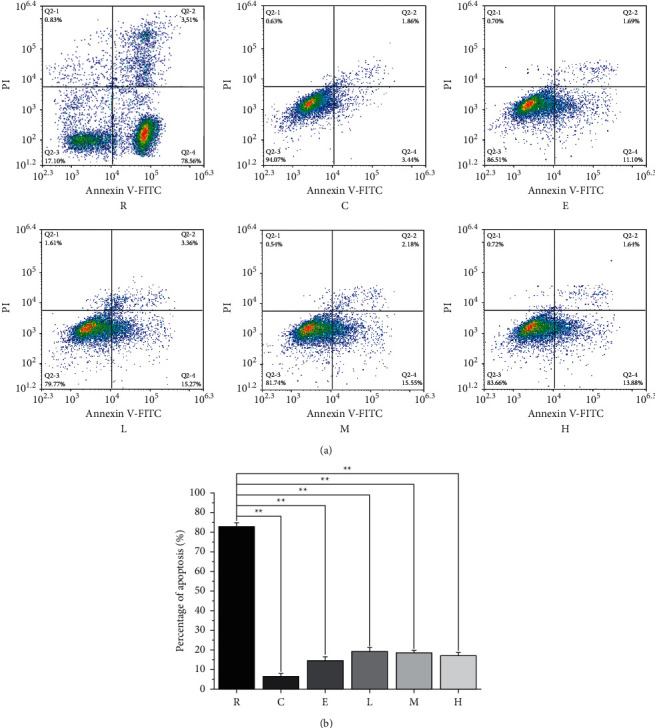
Detection of PC12 cells apoptosis after recovery from OGD. (a). Flow cytometry scatter plots. (b) Percentage diagram of apoptosis. ^*∗∗*^ means *p* < 0.01. Data are presented in the form of means ± standard deviation (*n* = 3). Control group (C); recovery from OGD group (R); edaravone group (E); NTE low-dose group (L); NTE middle-dose group (M); NTE high-dose group (H).

After recovery from OGD, PC12 cells were treated in different ways to detect apoptosis state. The results showed that the positive drug edaravone and different concentrations of NTE could significantly inhibit the apoptosis of PC12 cells. The effect of NTE on PC12 cells was concentration-dependent (Figure 2).

### 3.3. The Effect of NTE on the Cerebral Infarct Volume after CIRI

**Figure 3:**
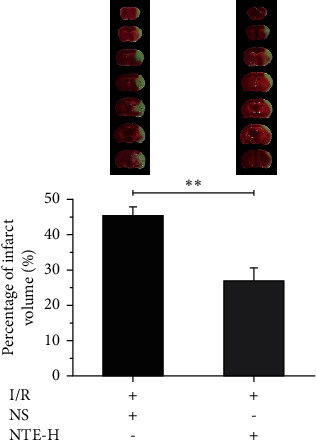
Measurement of infarct volume after CIRI. The upper part of the figure shows TTC staining of brain slices, with NS on the left and NTE-H on the right. The lower part is the percentage of infarct volume. ^*∗∗*^, + and − mean *p* < 0.01, treatment and no treatment, respectively. Data are presented in the form of means ± standard deviation (*n* = 5).

The infarct volume after CIRI was measured by TTC. The results showed that the infarct volume in normal saline group (NS) was significantly larger than that in NTE high-dose group (NTE-H), which indicated that NTE had protective effect after CIRI (Figure 3).

### 3.4. The Effect of NTE on Neuronal Apoptosis after CIRI

**Figure 4:**
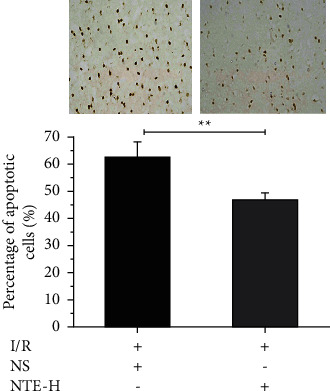
Detection of neuronal apoptosis after CIRI. The upper part of the figure shows TUNEL detection of cerebral cortex in brain slices, with NS on the left and NTE-H on the right. The lower part is the percentage of apoptotic cells. ^*∗∗*^, +, and − mean *p* < 0.01, treatment, and no treatment, respectively. Data are presented in the form of means ± standard deviation (*n* = 5).

TUNEL was used to detect neuronal apoptosis after CIRI. The results showed that NTE high-dose could significantly inhibit neuronal apoptosis in rat cerebral cortex (Figure 4), which indicated that NTE could realize neuroprotection by alleviating neuronal apoptosis after CIRI.

### 3.5. Screening and Identification of Differential Metabolites between NTE-P and NS-P

**Figure 5:**
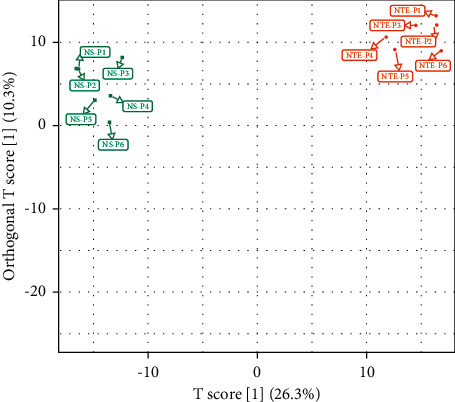
Plot of OPLS-DA scores. Green square represents NS-P group, and orange circle represents NTE-P group.

OPLS-DA was used to judge whether there are differences between NTE-P and NS-P. The results showed that NTE-P and NS-P could be clearly distinguished, and the internal correlation of each group was high (Figure 5).

**Figure 6:**
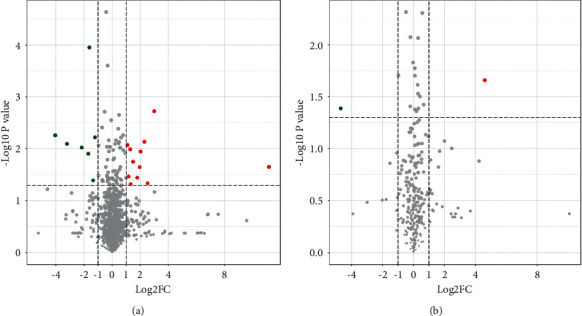
Volcanic map of differential metabolites. (a) The volcanic map of differential metabolites between NTE-P and NS-P in cationic mode. (b) The volcanic map of differential metabolites between NTE-P and NS-P in anionic mode. The red dot represents significantly upregulated metabolites (FC ≥ 2, *p* < 0.05), while the green dot represents significantly downregulated metabolites (FC ≤ 0.5, *p* < 0.05).

The differential metabolites were screened and identified according to the corresponding conditions. The results showed that compared with NS-P, NTE-P had 21 differential metabolites, of which 13 were significantly upregulated and eight were significantly downregulated (Figure 6 and Table 2). The representative substances of Astragalus root and Rhizoma Chuanxiong, calycosin and ferulic acid, existed in the differential metabolites, which indicated that the absorption of related substances in NTE into blood was an important reason for the difference in serum metabolites between NS-P and NTE-P.

### 3.6. KEGG Pathway Enrichment Analysis of Differential Metabolites

**Figure 7:**
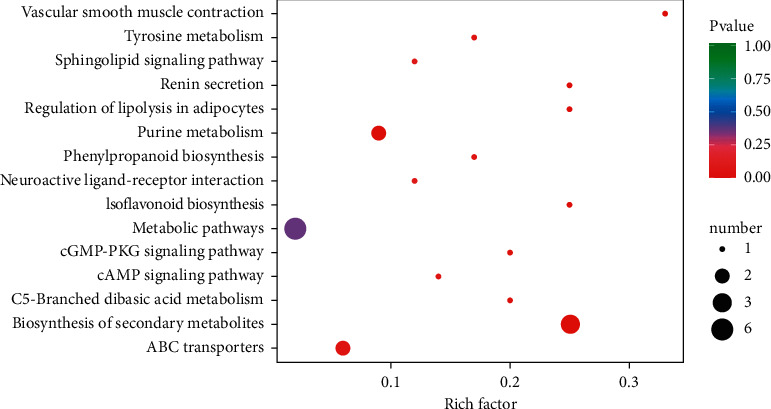
Enriched KEGG pathways. The color of the dot is *P* value. The closer to red, the more significant the *P* value. The size of the dot represents the number of differential metabolites. The larger the diameter is, the more the number is.

In order to further explore the biological function of differential metabolites, pathway enrichment analysis was carried out for all differential metabolites. As shown in Table 3 and Figures 6 and 7, the 21 different metabolites could be enriched in 15 metabolic processes, of which adenosine was involved in many metabolic processes and diverse physiological functions. It could be inferred from these results that NTE components absorbed into blood achieved neuroprotection by regulating various metabolic processes in the body, especially metabolic and physiological processes related to adenosine.

## 4. Discussion

In this study, the neuroprotective effect of NTE on alleviating neuronal apoptosis after CIRI was evaluated at both cell and tissue levels. On this basis, the serum samples from two groups of rats were detected by LC-MS. Meanwhile, differential metabolites and related metabolic pathways were screened and identified by multivariate statistical metrology. As a result, a total of 21 differential metabolites were identified. Through analyzing their related functions, metabolic pathways or signal pathways, our understanding about NTE inhibiting neuronal apoptosis and exerting neuroprotection could be further deepened.

### 4.1. Adenosine and Inosine

The metabolic pathway involved in the differential metabolites, adenosine and inosine, is purine metabolism, which has been proved to play a significant role in the CIRI [17]. Adenosine is an important neuromodulator, which not only regulates Alzheimer's disease, Parkinson's disease, epilepsy, inflammation, cancer, and other diseases but also alleviates the injuries caused by cerebral ischemia and reperfusion [18]. Previous studies had shown that exogenous injection of adenosine could upregulate the expression of A2A receptor in hippocampal cells after cerebral ischemia and reperfusion in rats, thus reducing the death of neurons in the hippocampal CA1 region and promoting the recovery of sensorimotor function [19]. Ganesana and Venton monitored release of adenosine during cerebral ischemia and reperfusion. The results showed that the release of adenosine increased instantaneously during cerebral ischemia and continued to increase until reperfusion [20]. Similar to the previously mentioned result, Wang and Venton studied the change of endogenous adenosine concentration during cerebral ischemia-reperfusion and its relationship with local blood-oxygen supply. The results showed that endogenous adenosine concentration increased significantly, which was positively correlated with local blood-oxygen supply [21]. In addition, Gholinejad et al. found that adenosine could reduce oxidative stress by inhibiting Mst1 expression and protect neural stem cells treated with H_2_O_2_ from apoptosis [22]. Inosine is a natural nucleoside derived from the degradation of adenosine, which is initially thought to have no biological effect. However, with the development of related research, the more and more results showed that inosine had effective immunoregulatory and neuroprotective effects. For example, Hsiao et al. reported that inosine could significantly inhibit platelet aggregation induced by agonists, thus alleviating cerebral ischemia and neuronal death [23]. Deng et al. also studied the effect of inosine on neuronal apoptosis in neonatal rats with cerebral ischemia injury. The results showed that inosine reduced the number of neuronal apoptosis by downregulating the expression of cytochrome C, thus achieving the neuroprotective function [24, 25]. All of the previously mentioned results fully showed that adenosine and inosine could use different ways to reduce neuronal death, especially programmed death dominated by apoptosis, in the process of CIRI. Accordingly, a key factor for NTE to inhibit neuronal apoptosis after CIRI in this study is that NTE could play a neuroprotective role by regulating the production of adenosine and inosine in purine metabolism.

### 4.2. Ferulic Acid and Calycosin

Ferulic acid and calycosin are two representative substances derived from Rhizoma Chuanxiong and Astragalus root, respectively. In this study, their presence was detected by metabonomics, indicating that they were important NTE constituents absorbed into the blood. At present, many studies have proved that ferulic acid plays a neuroprotective role in the process of CIRI. For instance, Deng et al. and Lin et al. had confirmed the protective effect of ferulic acid on CIRI from the whole and tissue level of animals, respectively [26, 27]. Yao et al. also proved the protective effects of sodium ferulate on ischemia and hypoxia of PC12 cell at the cellular level [28]. Further research on the neuroprotective mechanism showed that ferulic acid achieved neuroprotection in many ways, one of which is to inhibit neuronal apoptosis. Ren et al. found that ferulic acid played a neuroprotective role in CIRI through antioxidant and antiapoptotic mechanisms in vivo and in vitro [29]. Besides, Cheng et al. showed that ferulic acid could achieve neuroprotective effect through two antiapoptotic signal pathways: protein kinase B (Akt)/mammalian target of rapamycin (mTOR)/4E (eIF4E)-binding protein 1 (4E-BP1)/B-cell lymphoma-2 (Bcl-2) and p38 mitogen-activated protein kinase (p38 MAPK)/phosphorylates 90 kDa ribosomal S6 kinase (p90RSK)/cyclic AMP response element binding protein (CREB)/Bcl-2 [30, 31]. For calycosin, relevant studies could also prove its inhibitory effect on neuronal apoptosis after cerebral ischemia at the holistic, tissue, and cellular levels [32–34]. The results reported in literature explain a problem that NTE may play a significant role in the clinical therapy of CIRI by inhibiting neuronal apoptosis through ferulic acid and calycosin.

### 4.3. Salidroside, 6-Gingerol, and 2-Methoxycinnamaldehyde

Salidroside, 6-gingerol, and 2-methoxycinnamaldehyde were not representative substances of Rhizoma Chuanxiong or Astragalus root, but they were found in the NTE-P by metabolomics in this study, indicating that some substances contained in NTE could be further metabolized into other substances after being absorbed into the blood. Salidroside is a multifunctional bioactive substance, firstly found in Rhodiola. Relative studies had shown that salidroside had significant preventive and therapeutic effects on CIRI, of which the key mechanism was antineuronal apoptosis [35]. 6-gingerol and 2-methoxycinnamaldehyde are common in ginger and cinnamon. Although there was no direct evidence to prove that they played a role in the process of CIRI, studies on the role of other organs had been reported. For example, 6-gingerol could inhibit apoptosis by activating phosphoinositide 3-kinase (PI3K)/Akt pathway and high-mobility group box2 (HMGB2)-c-Jun NH2-terminal kinase1/2 (JNK 1/2)-nuclear factor-kappaB (NF-*κ*B) pathway, thus alleviating myocardial ischemia/reperfusion injury [36, 37]. 2-methoxycinnamaldehyde could alleviate hepatic ischemia/reperfusion injury through anti-inflammatory, antioxidant, and antiapoptotic effects [38]. Although the physiological activities exhibited by the previously mentioned three substances could not directly explain the protective mechanism of NTE against neuronal apoptosis, they suggested that NTE constituents might be transformed into other new substances in blood, which achieved the protective function of brain neurons by stimulating related metabolic pathways or signal pathways in the body.

## 5. Conclusions

NTE plays the neuroprotective effect by alleviating neuronal apoptosis after CIRI. The pharmacodynamic substances of this protective effect on the one hand are related to ferulic acid, calycosin, salidroside, 6-gingerol, and 2-methoxycinnamaldehyde in the blood components of NTE and on the other hand are also inseparable from NTE regulating purine metabolism to produce adenosine and inosine. Accordingly, NTE is a TCM compound with significant curative effects on CIRI.

## Figures and Tables

**Table 1:** Processing protocols of PC12 cells.

Grouping name	C	R	E	L	M	H
Glucose medium without CoCl_2_	+	−	−	−	−	−
Glucose-free medium containing 300 *μ*M CoCl_2_	−	+	+	+	+	+
After incubation for 8 h, the culture medium was changed as following protocols.						
Glucose medium containing 10% NS serum	+	+	−	−	−	−
Glucose medium containing 10% E serum	−	−	+	−	−	−
Glucose medium containing 10% NTE-L serum	−	−	−	+	−	−
Glucose medium containing 10% NTE-M serum	−	−	−	−	+	−
Glucose medium containing 10% NTE-H serum	−	−	−	−	−	+
After incubation for 48 h, the cells were treated according to the subsequent steps.						

“+” means processing; “−” means not processing.

**Table 2:** The information of differential metabolites.

Metabolite serial number	Metabolite	Molecular weight	Retention time (min)	Metabolite type	Difference situation
P1	PE-NMe (16 : 0/18 : 1 (11Z))	731.5465	10.123	Lipids and lipid-like molecules	Down
P2	Salidroside	300.1209	1.8754	Organic oxygen compounds	Up
P3	Stearoyl sphingomyelin	1507.1444	9.8363	Lipids and lipid-like molecules	Down
P4	Adenosine	267.0968	1.7143	Nucleotide And Its metabolomics	Up
P5	Inosine	268.081	1.3541	Nucleotide And Its metabolomics	Up
P6	PE-NMe (18 : 3 (6Z, 9Z, 12Z)/20 : 1 (11Z))	781.5622	7.6184	Lipids and lipid-like molecules	Down
P7	PC (20 : 0/14 : 1 (9Z))	637.5362	8.9476	Lipids and lipid-like molecules	Down
P8	(S)-2-Hydroxy-2-methylsuccinic acid	148.0372	9.6722	Lipids and lipid-like molecules	Up
P9	4-Nitrophenol	139.0269	12.9026	Benzene and substituted derivatives	Down
P10	N-Methyl-2-oxoglutaramate	159.0532	9.6706	Organic acids and derivatives	Up
P11	Gln Val Leu Leu Gly	528.3271	8.8566	Polypeptide	Up
P12	6-Gingerol	294.1831	9.7352	Benzenoids	Up
P13	PE-NMe (20 : 0/16 : 0)	761.5935	11.7553	Lipids and lipid-like molecules	Down
P14	2-Methoxy-cinnamaldehyde	162.0681	9.6706	Phenylpropanoids and polyketides	up
P15	Val Cys Leu	333.1722	1.3978	Polypeptide	Down
P16	Diisobutyl phthalate	278.1518	9.6737	Phenolic acids	Up
P17	Calycosin	284.2635	8.6701	Organic oxygen compounds	Up
P18	Artomunoxanthentrione	1437.0661	10.7763	Organoheterocyclic compounds	Up
P19	Ferulic acid	194.184	12.5372	Phenylpropanoids and polyketides	Up
P20	5-Methylthio-D-ribose	180.0456	2.35	Organic oxygen compounds	Down
P21	Cytidine-5'-monophosphate-5-N-acetylneuraminic acid	614.1473	1.3641	Nucleosides, nucleotides, and analogues	Up

**Table 3:** Corresponding differential metabolites in the enriched KEGG pathways.

KEGG number	Metabolic pathway	Metabolite serial number
Ko00230	Purine metabolism	P4, P5
Ko00350	Tyrosine metabolism	P2
Ko00660	C5-Branched dibasic acid metabolism	P8
Ko01100	Metabolic pathway	P2, P4, P5, P8
Ko02010	ABC transporters	P4, P5
Ko04022	cGMP-PKG signaling pathway	P4
Ko04024	cAMP signaling pathway	P4
Ko04071	Sphingolipid signaling pathway	P4
Ko04080	Neuroactive ligand-receptor interaction	P4
Ko04270	Vascular smooth muscle contraction	P4
Ko04923	Regulation of lipolysis in adipocytes	P4
Ko04924	Renin secretion	P4
Ko00940	Phenylpropanoid biosynthesis	P19
Ko00943	Isoflavonoid biosynthesis	P17
Ko01110	Biosynthesis of secondary metabolites	P2, P17, P19

## Data Availability

The data used to support the findings of this study are available from the corresponding author upon request.
